# Stabilizing milk-derived extracellular vesicles (mEVs) through lyophilization: a novel trehalose and tryptophan formulation for maintaining structure and Bioactivity during long-term storage

**DOI:** 10.1186/s13036-024-00470-z

**Published:** 2025-01-13

**Authors:** Alan B. Dogan, Spencer R. Marsh, Rachel J. Tschetter, Claire E. Beard, Md R. Amin, L. Jane Jourdan, Robert G. Gourdie

**Affiliations:** 1 Virginia Tech Carilion School of Medicine, Roanoke, VA 24016 USA; 2 Fralin Biomedical Research Institute at Virginia Tech Carilion, Roanoke, VA 24016 USA; 3 Center for Vascular and Heart Research, Virginia Tech, Roanoke, VA 24016 USA; 4 Department of Biomedical Engineering and Mechanics, Virginia Tech, Blacksburg, VA 24061 USA; 5 Department of Emergency Medicine, Virginia Tech Carilion School of Medicine, Virginia Tech, Roanoke, VA 24016 USA; 6 Faculty of Health Science, Virginia Tech, Blacksburg, VA 24061 USA; 7 Translational Biology, Medicine, and Health graduate program at Virginia Tech, Roanoke, VA 24016 USA; 8 Materials Science and Engineering, Virginia Tech, Blacksburg, VA 24061 USA

**Keywords:** Milk-derived EVs, Extracellular vesicles, Lyophilization, Trehalose, Tryptophan, Room temperature storage, Freeze-drying, Drug delivery systems, Bioactivity preservation

## Abstract

Extracellular vesicles (EVs) are widely investigated for their implications in cell-cell signaling, immune modulation, disease pathogenesis, cancer, regenerative medicine, and as a potential drug delivery vector. However, maintaining integrity and bioactivity of EVs between Good Manufacturing Practice separation/filtration and end-user application remains a consistent bottleneck towards commercialization. Milk-derived extracellular vesicles (mEVs), separated from bovine milk, could provide a relatively low-cost, scalable platform for large-scale mEV production; however, the reliance on cold supply chain for storage remains a logistical and financial burden for biologics that are unstable at room temperature. Herein, we aim to characterize and engineer a freeze-dried, mEV formulation that can be stored at room temperature without sacrificing structure/bioactivity and can be reconstituted before delivery. In addition to undertaking established mEV assays of structure and function on our preparations, we introduce a novel, efficient, high throughput assay of mEV bioactivity based on Electric Cell Substrate Impedance Sensing (ECIS) in Human dermal fibroblast monolayers. By adding appropriate excipients, such as trehalose and tryptophan, we describe a protective formulation that preserves mEV bioactivity during long-term, room temperature storage. Our identification of the efficacy of tryptophan as a novel additive to mEV lyophilization solutions could represent a significant advancement in stabilizing small extracellular vesicles outside of cold storage conditions.

**Supplementary Information:**

The online version contains supplementary material available at 10.1186/s13036-024-00470-z.

## Background

Extracellular vesicles (EVs) are a heterogeneous group of cell-derived membranous structures, ranging from nanometers to micrometers in size, which are released into the extracellular environment by a variety of eukaryotic and prokaryotic cells [1–3]. Comprising exosomes (30–150 nm), microvesicles, and apoptotic bodies (> 1000 nm), each with distinct biogenesis pathways, EVs are produced endogenously by nearly every mammalian organism and serve as key cellular messengers; delivering cargo and chemical signaling to modulate recipient cell responses [4]. Small and large EVs, which are < 200 nm and 200-1000 nm, respectively, have gained increasing interest as biomarkers, drug delivery vectors for lipid, protein and nucleic acid payloads and as a standalone therapeutic [5–7].

While EVs have recently gained popularity as a new therapeutic platform [8–10], two significant hurdles exist for adaptation of EVs from benchtop to bedside; scalable, high purity isolation and cost-efficient, long-term storage [11–13]. Over the past decade, it has been discovered that mammalian milk contains a particularly high concentration of EVs [7, 14], which presents as a potential solution to scalable isolation. Our group is investigating milk-derived extracellular vesicles (mEVs) as a potential therapeutic and as a drug delivery vector for peptide therapeutics. We have previously demonstrated that raw bovine milk can produce structurally and functionally intact mEVs that can be separated from the inherent macromolecules in milk [15].

Nevertheless, EVs lose efficacy and structural integrity over time from thermodynamic stress, shear stress, oxidative stress, and chemical degradation [16–18]. Thermodynamic-induced EV damage has been relatively understudied, and many groups generally accept that short-term storage at 4ºC and long-term storage at -20ºC to -80ºC are acceptable conditions [19–21]. However, when translating an EV product to the clinic or market, this creates a reliance on the cold supply chain, which has proven to be costly and incompatible for delivering immediate care [22, 23]. Additionally, freeze-thaw cycling has been shown to have various undesirable effects on EV structure [17, 24, 25], however the impact on EV bioactivity and internal cargos is largely unknown.

To address the challenges related to cold storage, various groups have explored methods for stabilizing EVs in both aqueous and solid formulations [26–28]. Among these approaches, lyophilization, or freeze-drying, has gained prominence as an effective means to preserve EVs and exosomes at room temperature, particularly using cryoprotectants and bulking agents like trehalose (TH), sucrose, mannitol, and amino acids [29–32]. Although general formulations of TH and other excipients have been explored, the concentrations vary depending on the source of EVs (e.g., cancer-derived, mesenchymal-derived, or serum-derived), and many studies do not evaluate the in vitro bioactivity of EVs before and after lyophilization [28]. Therefore, further research is needed to assess the overall stability of mEVs before and after lyophilization, with the goal of improving mEV functionality during long-term storage.

Herein, we introduce a freeze-dried mEV formulation designed for long-term storage at room temperature without compromising the structure and function of mEVs. Of particular note, we identified that addition of 100 µM tryptophan as a lyophilizate excipient resulted in significant improvements in structural and functional parameters measured from mEVs that were reconstituted in aqueous solutions following freeze-drying. Ultimately, advancement in mEV handling sets the stage to potentially eliminate the need for cold storage, overcoming distribution bottlenecks for future EV therapeutics.

## Materials and methods

### Milk-derived extracellular vesicle isolation

mEV isolation from bovine milk was achieved according to a previously established protocol [15]. Briefly, unpasteurized milk was defatted via centrifuged twice at 2,500 rcf for 30 min, and 4 times at 22,600 rcf. Skimmed milk was then filtered consecutively with 0.45 μm and 0.22 μm filters. The solution was then chelated with 30 mM EDTA at 37ºC for 1 h before undergoing tangential flow filtration (TFF) at 15 mL/min with 10x diafiltration. Filtrate was then separated with an IZON qEV original 70 nm sepharose column using HEPES buffer (20 µM HEPES, 100 µM NaCl, 4 mM KCl, pH 7.4, sterile/degassed under room atmosphere) as eluent. mEV containing fractions with minimal endogenous protein were collected.

### Lyophilized mEV Preparation and Loading

Purified mEV fractions mixed with cryoprotectants and bulking agents (e.g., trehalose, sucrose, tryptophan) at titrated concentrations (1 µM – 100 mM) according to previously reported protocols [18, 26, 33, 34]. Solution volumes of 0.2–5 ml were freeze-dried as follows. Samples were placed on ice and subsequently frozen under controlled temperatures. Samples were then added to the lyophilizer (Advantage Plus EL85, VirTis). Lyophilization vacuum and temperature was controlled using equipment guidelines. During “standard lyophilization” samples were frozen on dry ice (-70ºC) for 5 min and placed on maximum vacuum (~ 10 mTorr), standard shelf temperature (20ºC), and minimum condenser temperature (-96ºC) (Figure S1a). Lyophilized samples for characterization and in vitro testing were stored at room temperature under vacuum and reconstituted in room temperature diH_2_O equal to the volume of the mEVs originally isolated [17, 35–37].

### Zeta Potential / dynamic light scattering (DLS)

Particle surface charge has previously used as a proxy for mEV membrane stability. Sample zeta potentials analysis was performed on a Zetasizer Nano ZS (Malvern). mEV samples were diluted 1:10 in HEPES buffer, then underwent bath sonication in a Branson 2510 bath sonicator (30 s at RT) to reduce sample aggregation. Samples were then loaded into the Zetasizer and surface charge (mV) was measured in triplicates. Similarly, samples were analyzed on the Zetasizer Nano ZS using DLS.

### Nanoparticle tracking analysis (NTA)

To assess the size distribution of mEVs, Nanoparticle Tracking Analysis (NTA) was performed on a NanoSight NS300 (Malvern Panalytical, Malvern, UK) at RT. mEVs samples were diluted 1:10 in degassed HEPES buffer, then bath sonicated (30 s at RT) to reduce mEV aggregation. mEVs were then diluted 1:1,000 or 1:10,000, set on a syringe pump (Malvern Panalytical) and loaded into the NanoSight large volume flow cell with a flow rate of 0.003 mL/min.

According to previous studies, each sample was analyzed using a 405 nm laser with 5 consecutive 1-minute video recordings with a constant flow rate set at 10 U [38, 39]. All videos were compiled and analyzed in the NTA software (Version 3.4). Results are displayed as the sample mean (black bar), mode (yellow dot), and standard deviation (error bars) or, in some cases, as a particle distribution histogram. Polydispersity index (PDI) was calculated using the following equation:

$$\:PDI=\frac{{\text{S}\text{t}\text{a}\text{n}\text{d}\text{a}\text{r}\text{d}\:\text{D}\text{e}\text{v}\text{i}\text{a}\text{t}\text{i}\text{o}\text{n}}^{2}}{{\text{S}\text{a}\text{m}\text{p}\text{l}\text{e}\:\text{M}\text{e}\text{a}\text{n}}^{2}}$$

### Transmission Electron Microscopy (TEM)

TEM was performed to characterize mEV shape and size and to verify NTA size distribution results. TEM samples were prepped according to a previously established protocol [15]. Briefly, formvar-coated 200 mesh copper grids (Electron microscopy sciences, Hatfield PA, FCF200-CU) were glow discharged on a Pelco glow discharge unit (Pelco, Fresno CA) at 0.29 mBAR for 1 min. 0.1% poly-L-Lysine was applied to the grid for 1 min and excess solution wicked away with Whatman (Whatman PLC, Maidstone UK) #1 filter paper. Grids were washed 2x with 10 µL milli-Q and dried grids were loaded with 10 µL of prepared mEV concentrate and allowed to incubate for 5 min. Samples were then negatively stained with 10 µL Uranyless stain (Electron microscopy sciences, 22409) for 1 min at RT. Grids were allowed to dry overnight at RT before transmission electron microscope (TEM) imaging. Imaging of was performed on a FEI Tecnai G20 Biotwin TEM (FEI Company, Hillsboro OR) at 120 kV and images were captured using an Eagle (GATAN, Pleasanton, CA) 4 K HS camera. mEV diameters were obtained manually using ImageJ.

### Differential scanning calorimetry (DSC)

To determine the glass transition temperature (T_g_) and melting point (T_m_) of lyophilized mEV samples, differential scanning calorimetry (Setaram Inc., Virginia Tech Dept. of Materials Science & Engineering) was performed. Lyophilized samples were weighed (2–10 mg), placed in the DSC’s crucible, and measured under a N_2_ atmosphere with a heating rate of 10 K/min. Differential thermograms were analyzed using Advantage/Universal Analysis (UA) software (TA Instruments). Error bars represent instrument precision approximated to 5% error.

### ECIS bioactivity wound healing assay

Due to the inherent diversity in EV synthesis, structure, and function, there is not a standardized in vitro bioactivity assay to assess EV bioactivity. However, mEVs supplementation to cell culture have been previously shown to enhance wound healing by augmenting cell-cell signaling [40, 41]. Specifically, our group has found that on scratch-assays, a mEV dose of 21 µg/mL (~ 19–21 µL of mEVs per 400µL well, adjusted for protein concentration post-SEC) had a measurable effect on cellular migration [42]. Therefore, we chose to leverage electric cell-substrate impedance sensing (ECIS) instrumentation (Applied Biophysics, NY, USA) to monitor human dermal fibroblast (HuDF) migratory response after an induced electrical injury, similar to a traditional scratch-assay [43–49]. Briefly, 8W1E or 96W1E electrode ECIS arrays were coated with 10mM L-cysteine, followed by 1% gelatin according to manufacturer guidelines. Electrodes were then stabilized in HuDF complete media, and subsequently seeded with HuDF cells at a density of 1 × 10^5^ cells/well in a final volume of 400 µL. Cells were incubated for ~ 6–10 h to achieve ~ 80% confluency, which was confirmed by previously performed growth-phase profiles. A 25 kHz, 2600 µA, 20 s electric wound was then applied for cells and resistance was monitored at 4000 Hz until resistance values stabilized, signifying completed cell migration. Cell migration completion time (t_recovery_) was determined by a custom Python script that determined when resistance values stabilized in each well. Rate of recovery post-wound (t_control_ / t_group_) is normalized using controls from each individual trial, setting controls to a value of 1.

### Gel electrophoresis and western blotting

Gel Electrophoresis and Western blot (WB) analysis was performed as previously described [15]. Samples were separated by sodium dodecyl sulfate polyacrylamide gel electrophoresis (SDS-PAGE) and transferred to a PVDF (MilliporeSigma, St. Louis MO, IPFL00010) membrane. Membranes were blocked in EveryBlot Blocking Buffer (Bio-Rad Laboratories, Hercules CA, cat. # 12010020) for 5 min at room temperature. Overnight primary antibody incubation was performed and primary antibodies were diluted in the blocking buffer as follows: CD9 (Novus Biologicals, Littleton CO, NB500-494, 1:1000), Calnexin (MilliporeSigma, Burlington MA, AB2301, 1:5000), TSG101 (Bethyl Laboratories Inc. Montgomery TX, A303-506 A, 1:5000). Following washing, the membrane was incubated for 1 h at room temperature with secondary antibodies diluted 1:20,000 for mouse (Jackson ImmunoResearch, West Grove, PA, 715-035- 150) and 1:20,000 for rabbit (Southern Biotechnology, Birmingham, AL, 4050-05) in blocking buffer. Proteins of interest were visualized by chemiluminescence using a Bio-Rad ChemiDoc MP imager.

### CTDR-loaded mEV targeting and Calcein-AM imaging

mEV stability and cellular uptake experiments, mEVs were loaded with Calcein-AM dye (Thermo Scientific, C1430) or Cell Tracker Deep Red (CTDR) (Thermo Scientific, C34565) using a previously established protocol [15]. Briefly, mEVs were incubated at 37 °C for 2 h in the presence of 20 µM of payload then centrifuged at 16,873 x g for 1 h at 4 °C to remove unincorporated dye. Supernatant was replaced with fresh HEPES and stored overnight at 4 °C. Samples are sterile filtered prior to being administered in both scratch assays and animal experiment sections below.

Biologic uptake ability of mEVs were assessed using Madine Darby Canine Kidney (MDCK) Cells with enhanced Cx43. Medium used for the MDCK cells was M199 (Millipore Sigma M4530) supplemented with 10% Fetal Bovine Serum (FBS; Thermo Fisher/Gibco, 26140-079) and 1% Hygromycin B (Sigma H0654). Cells were expanded and stored in liquid nitrogen until plating on standard polypropylene culture dishes in culture medium described above. Cells were expanded to confluency, then passaged into 12-well plates for analysis.

Samples were sterile filtered prior to being administered in both scratch assays and animal experiment sections below. Cells or tissues were processed as explained in sections below, then imaging is performed on a Leica TCS SP8. Cells were plated onto sterile coverslips within the plate, then given 2 days to adhere and grow prior to scratch assay. Assay was performed by using a 200 µL sterile pipette tip to gently scratch the surface of the cells, then cells were rinsed 1x in dPBS (Invitrogen, 14080055) and provided fresh culture medium supplemented with CTDR-tagged mEVs. Cells were given 15 min to take up mEV’s, then were rinsed in 1x dPBS and fixed in 2% Paraformaldehyde (Fisher O4042-500). Cells were rinsed 4x in PBS, then stained in 1:20,000 Hoescht (Life Technologies, H3569) and rinsed one additional time in PBS. Coverslips were then removed and adhered to microscope slide, and imaged on a Leica SP8. Images were analyzed in ImageJ by saving the individual red channel and converting to 8-bit, thresholding, and counting particle numbers. These were done in triplicate with 10 images taken of each sample for an individual experiment *n* = 30, with experiments being done in triplicate.

For assessing mEV membrane integrity, mEVs were incubated at 37 °C for 2 h in the presence of 10 µM of calcein-AM. After incubation, extravesicular dye was removed with Sepharose G50 spin columns (USA Scientific, Ocala FL 1415–1601) pre-equilibrated with HEPES buffer. 6 µL of loaded mEV solution was then dispensed onto a microscope slide (Premiere Scientific, Grand Prairie TX, 75 × 25 × 1 mm, 9105), cover-slipped (Thermo Scientific, 12541 A), and imaged on a Leica SP8 confocal microscope (Leica Camera AG, Wetzlar Germany) with 488 laser, HyD, 1AU, at scan frequency of 700 Hz for 6 fields per slide.

All reported intensity values represent the mean of all captured images on a log scale, with biological replicates.

### Statistics

Experiments were carried out over several batches of mEV isolations. When possible, comparisons were made within the same isolation batch. In vitro data represents the average of ECIS wells with error bars representing the standard error of the mean. DSC and NTAs were all carried out a single time for each batch, as these represent bulk properties of an isolate. Fluorescence studies were carried out in biological duplicates and log-scaled relative to fresh mEV controls (µ set to 1). Statistics, multiple T tests with Bonferroni Correction (α = 0.05), were carried out in Python using the *scipy* and *pandas* libraries.

## Results

### mEV isolation and baseline characterization

**Fig. 1:**
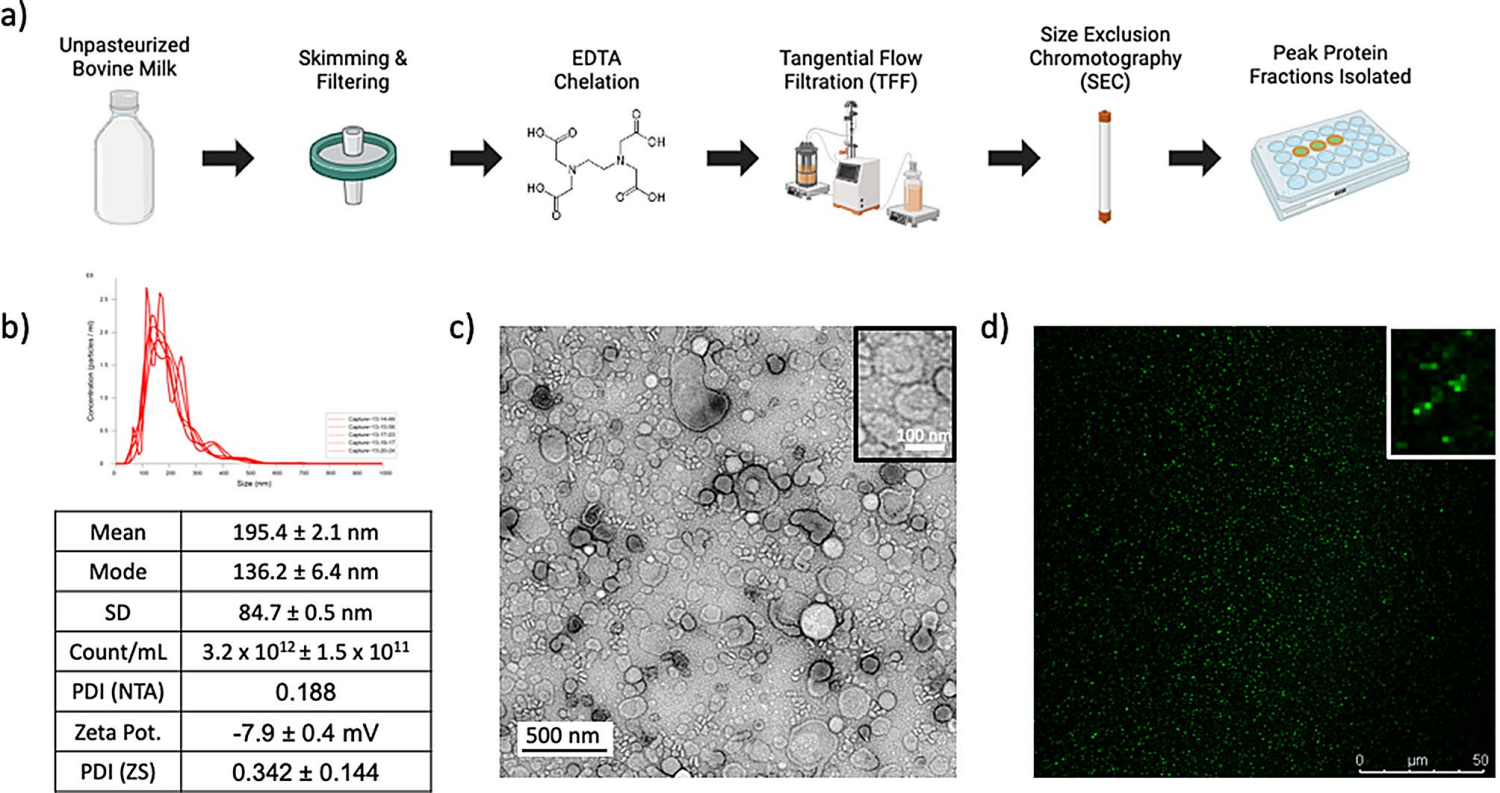
**(a)** Process overview of mEV isolation from unpasteurized bovine milk **(b)** NTA particle size distribution of freshly isolated mEVs, reported as mean, mode, standard deviation (SD), sample particle count (Count/mL), and calculated polydispersity index (PDI). Zetasizer obtained zeta potential (Zeta Pot.) and PDI (annotated ZS) (*n* = 3). **(c)** TEM image of mEVs (Mean: 126 nm, SD: 42 nm, *N* = 60 particles measured) with zoomed-in panel of isolated mEVs **(d)** Calcein-AM loaded mEVs under fluorescent microscopy

mEVs isolated from bovine milk showed similar characteristics to previously reported studies [15]. Peak protein fractions following SEC produced mEVs with a mean distribution varying between 160 and 190 nm, a mode varying between 130 and 160 nm, and a zeta potential of -7.9 ± 0.4 mV (Fig. 1b). TEM revealed minimal bovine milk proteins (e.g., casein) associated with mEVs, suggesting successful removal of milk proteins during EDTA chelation, TFF, and SEC, with a mean particle diameter of 126 nm (SD: 42.6 nm, 60 counts, ImageJ) (Fig. 1d). The top three mEV fractions from the final SEC step varied between 0.7 and 1.1 µg/mL, as determined by UV-Vis spectroscopy (280 nm). These fractions, as previously shown by our group, express EV markers CD9 and CD81 and do not express endoplasmic reticulum marker Calnexin and endosomal marker Arf6 [15].

Calcein-AM loaded mEVs were used to assess membrane integrity (Fig. 1e). Calcein-AM is de-esterified by esterase enzymes within the mEVs; high levels of punctate fluorescence and low background suggests retained esterase activity throughout isolation and retained membrane integrity throughout purified mEV handling.

### mEVs accelerate HuDF wound closure in an ECIS-based assay in vitro

**Fig. 2:**
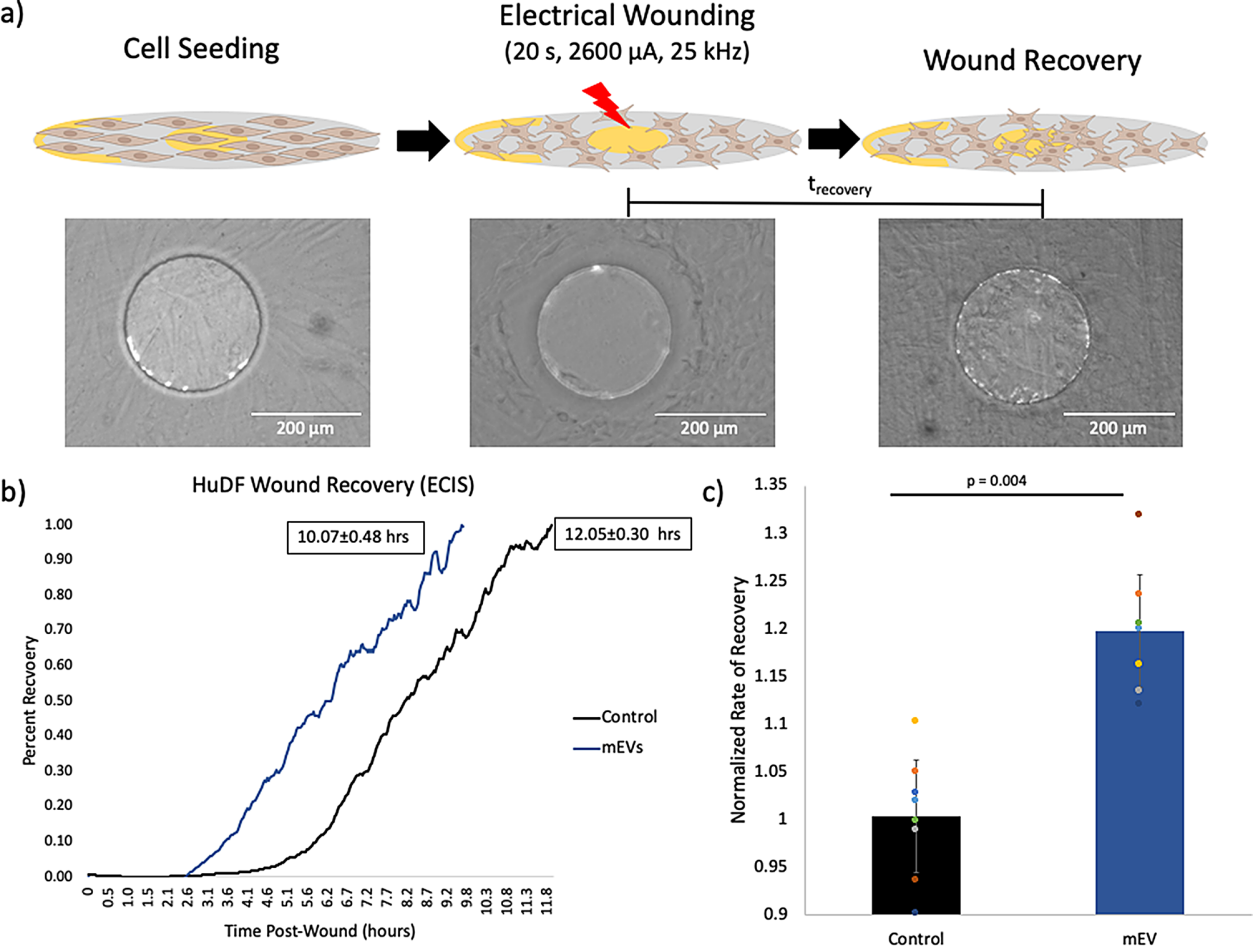
**(a)** Outline of ECIS electric-wound assay with bright field images of cell monolayers during seeding, wound induction, and recovery. **(b)** Representative time-series data of resistance (measured at 4000 Hz) normalized to total recovery time, controls recovered in 12.05 ± 0.30 h, mEV treated groups recovered in 10.07 ± 0.48 h **(c)** HuDF monolayer recovery rates normalized such that the no-treatment controls were set to 1. Two-sample t-test shows statistical significance between mEV treatment and controls (*p* = 0.00423), *N* = 8

EVs have been previously reported to enhance cell-cell interactions and accelerate wound healing in other applications [41, 50]; however, there is not a standardized way of assessing mEV bioactivity, especially after long-term storage. In this study, we employed Electric Cell-substrate Impedance Sensing (ECIS) to assess the resistance generated by a cell monolayer and to quantify the rate at which the monolayer recovers from a circular wound inflicted by an electrical shock. We found that a 21 µg/mL dose of fresh mEVs added to a cell media volume of 400 µL sped up the time to recover by ~ 20%, triggering monolayer recovery at 10.07 ± 0.48 h as opposed to 12.05 ± 0.30 h in control (Fig. 2b and c). Notably, time-series resistance data suggests that mEV-treated cells begin the recovery process within the first 2–3 h after wound induction; which is a two-hour improvement over controls (Fig. 2b).

### mEVs degrade during cold storage and after a single freeze-thaw cycle

**Fig. 3:**
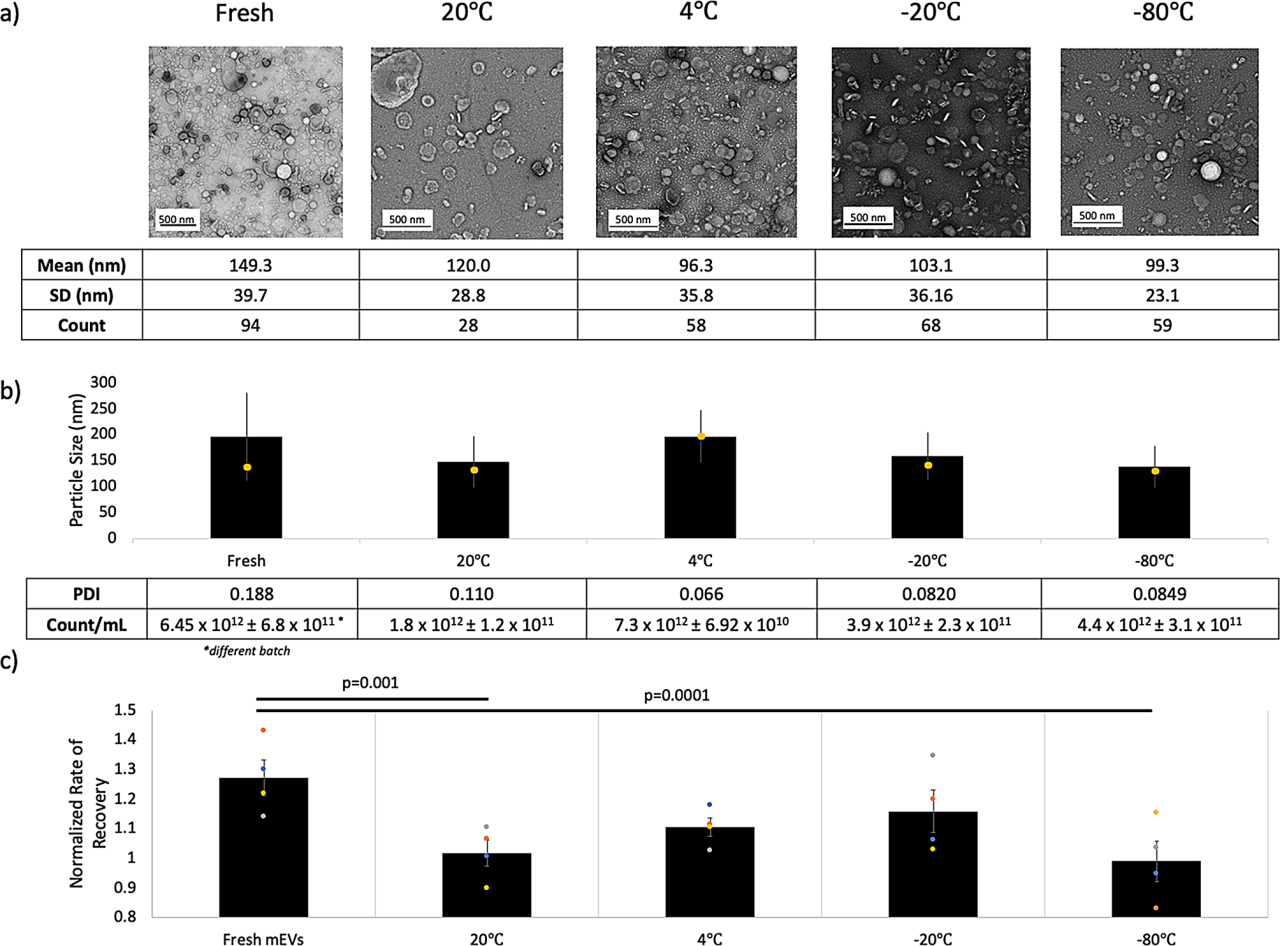
**(a)** TEM with manually measured mean, SD, and counts per frame **(b)** NTA particle distribution (mean {black bar}, mode {yellow marker}, STDEV {error bars}), calculated polydispersity index (PDI), and particle counts. Means were not statistically different via using multiple T tests with Bonferroni Correction (α = 0.05) **(c)** ECIS scratch-assay bioactivity for mEV samples stored for 1 month in various conditions in HEPES buffer. ECIS wound recovery was analyzed by multiple T tests with Bonferroni Correction (α = 0.05); statistically significant differences were noted between fresh mEVs and 20ºC (*p* = 0.001) and − 80ºC (*p* = 0.0001); *N* = 4

While it is widely known that isolated EVs do not remain stable for extended periods of time, there is little data characterizing the degradation of EVs under different storage conditions [17, 24, 51]. Zeta surface charges remained relatively constant among all temperature groups, suggesting that the mEV surface chemistry is unchanged by a single freeze-thaw cycle or storage at 20ºC and 4ºC (Figure S2a). However, TEM imaging revealed a notable decrease in observable particles after 1 month of storage under all storage conditions (Fig. 3a). Groups that underwent a freeze-thaw cycle had decreased particle counts compared to fresh controls, however particle size distributions remained statistically unchanged (Fig. 3b).

While mEV surface chemistry remained unchanged, bioactivity from our ECIS assay in HuDF monolayers differed significantly. It is widely reported that mEVs exert a dose-dependent effect on targeted cells [52]; however, even when each group was matched according to protein concentration, we observed statistically significant loss of HuDF activity amongst 20ºC and − 80ºC groups (Fig. 3c). Only 4ºC, -20ºC storage retained mEV bioactivity, despite slight variabilities in particle counts and PDI compared to fresh controls.

### Trehalose shields mEVs, preserving structure and function

Trehalose (TH) has been previously reported to preserve EV structure and internal cargo [26, 35, 29, 53]. However, the mechanism and concentration dependence of TH’s action remains unclear for mEVs.

**Fig. 4:**
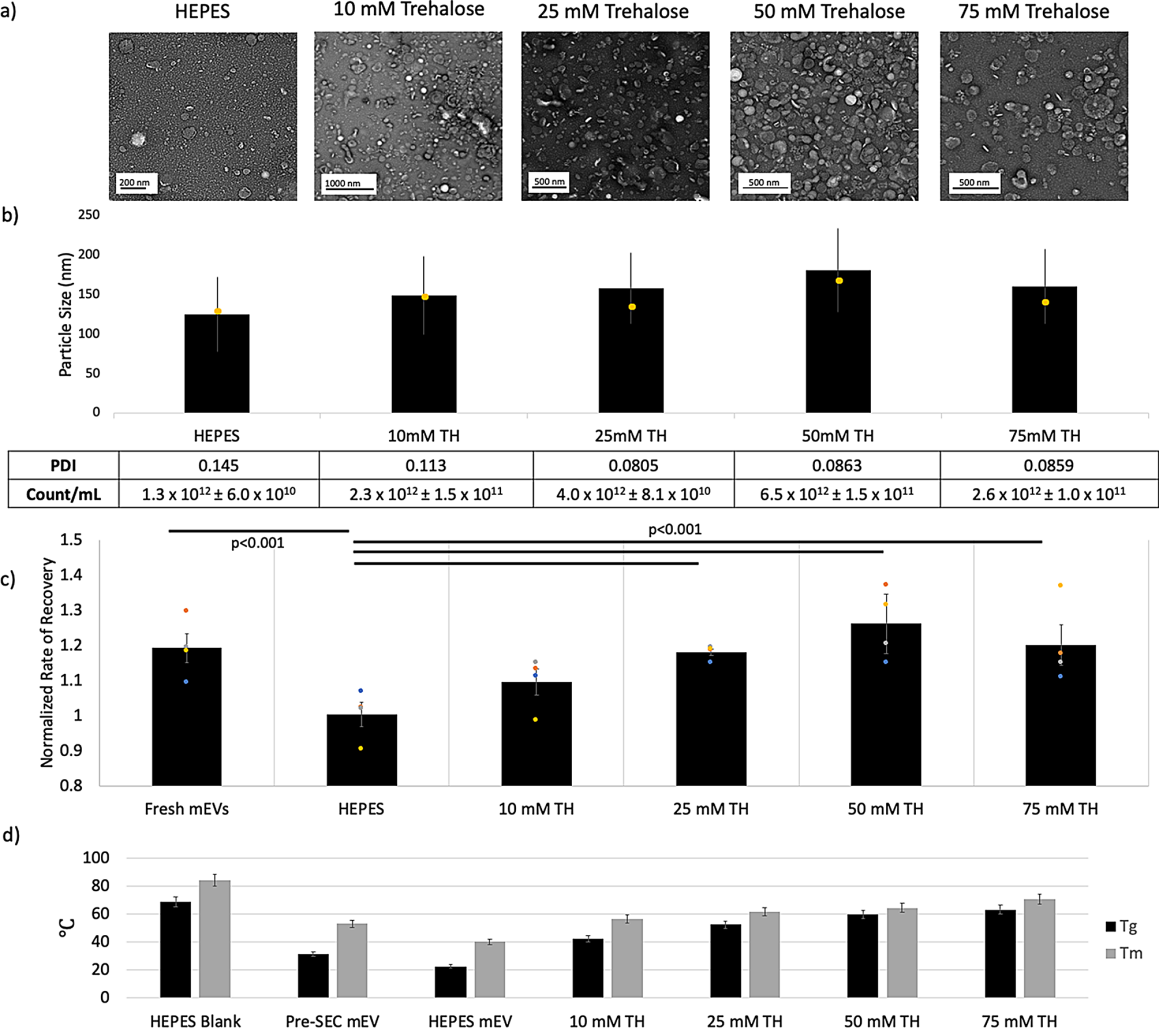
**(a)** TEM images of lyophilized mEV reconstituted in diH_2_O, **(b)** NTA particle distribution (mean {black bar}, mode {yellow marker}, STDEV {error bars), PDI, and particle counts. Means were not statistically significant using multiple T tests with Bonferroni Correction (α = 0.05) **(c)** ECIS assay bioactivity of lyophilized mEV samples stored for 1 month at room temperature in various concentrations of Trehalose (TH), *N* = 4. ECIS wound recovery was analyzed using multiple T tests with Bonferroni Correction (α = 0.05); there was significantly diminished bioactivity in HEPES-lyophilized samples compared to fresh mEVs and 25mM, 50mM and 75mM TH groups all had statistically greater bioactivity compared to HEPES-lyophilized controls **(d)** DSC thermal stability of mEV powders *N* = 1; error bars represent standard error associated with calibration. HEPES blanks were obtained by lyophilizing 5mL of neutral buffer. Pre-SEC mEVs were obtained by lyophilizing 5mL of eluent before concentrating. Thermograms of a few of the samples are available in Figure S7

mEVs lyophilized (procedure outlined in Figure S1a) in HEPES-alone resulted in destruction of mEVs, which is seen across all structure and function quality control parameters, including bioactivity (Fig. 4). 50mM TH showed highest retention of particle count and size along with retained bioactivity in vitro (Fig. 4a-c). Throughout all TH and sucrose groups, there is a relative loss of larger particles during the lyophilization step, resulting in left-shifted distribution in the nanoparticle size histograms on NTA (Fig. 4, S3). 25- (*p* = 0.0001), 50- (*p* = 0.0004) and 75-mM (*p* = 0.0017) TH groups statistically outperformed HEPES-only controls, which corresponds to the retained mEV structure seen on TEM and NTA (Fig. 4c). Fresh controls were only significantly different from HEPES-only lyophilized samples (*p* = 0.0004).

Thermal analysis revealed that the glass transition temperature (T_g_) of mEVs lyophilized in HEPES is ~ 20ºC, suggesting instability at room temperature. Pure TH had a high T_g_ of 96.7ºC, which aligned with previously reported values between 79 and 115 ºC [54, 55]. The addition of TH to mEV solutions resulted in a linear increase of both T_g_ and T_m_; indeed, the addition of 75 mM TH raised Tg from around 20ºC to 60ºC, and Tm from 40ºC to 70ºC. Isolated mEVs pre-SEC (i.e., before the removal of exogenous milk proteins) revealed a slightly higher thermal stability (31ºC and 53ºC; T_g_ and T_m_).

Other disaccharides, such as lactose and sucrose, were also investigated at 50mM concentrations, and it was found that TH was the top performer in both particle distribution and bioactivity (Figure S3).

### Trehalose and tryptophan synergistically interact with mEVs

To further ensure stability of freeze-dried biologic powders, low molecular weight excipients like amino acids and mannitol are commonly used as bulking agents [56, 57]. Physiologically, it has been found that in both human and bovine milk, the concentration of non-protein tryptophan increases in colostrum production soon after pregnancy within the ranges of 4–60µM [58–60]. In addition, lactadherin, a protein known to have high affinity to mEVs, has several tryptophan residues in its binding site pocket [61, 62]. Therefore, we hypothesized that tryptophan may exhibit favorable stabilizing properties toward mEVs while also serving as a bulking agent during lyophilization. Tryptophan concentrations of 10µM, 100µM, 1mM were chosen to cover physiological ranges present in colostrum and are dilute enough such that they do not cause a pH shift in HEPES buffers.

**Fig. 5:**
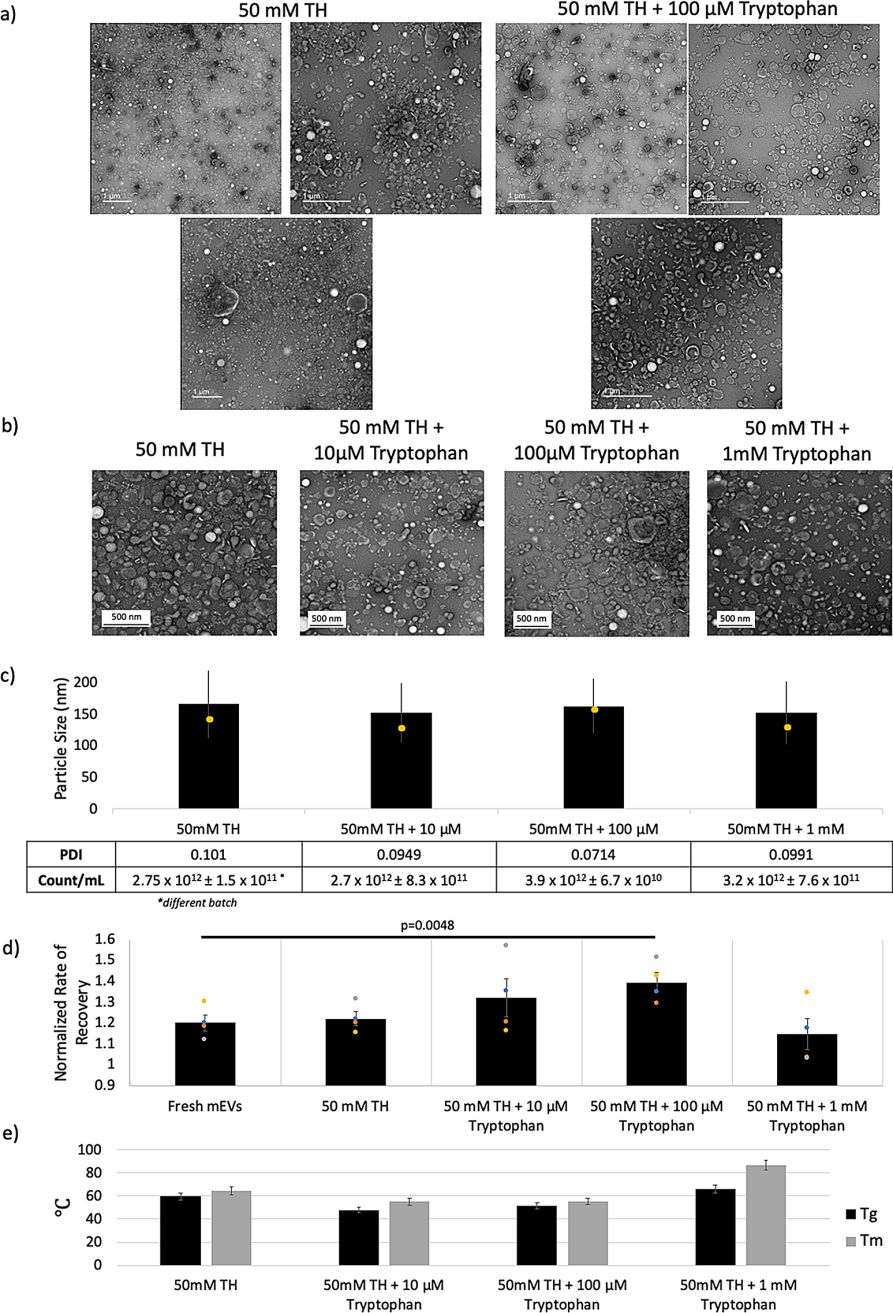
**(a)** TEM images of lyophilized mEVs stored in 50mM TH and 50mM TH + 100µM Tryptophan **(b)** TEM images of lyophilized mEVs reconstituted in diH_2_O **(c)** NTA particle distribution. All group means were not statistically significant (Multiple T tests with Bonferroni Correction, α = 0.05) **c)** ECIS scratch-assay bioactivity for lyophilized mEV samples stored for 1 month at RT. Multiple T tests with Bonferroni Correction (α = 0.05) showed statistical significance between fresh mEV controls and 50mM TH + 100µM Tryptophan (*p* = 0.0048); *N* = 4 **(d)** DSC thermal stability of mEV powders *N* = 1, error bars represent standard error associated with calibration

Qualitatively, TEM imaging showed that when comparing TH-only samples to samples supplemented with 100µM tryptophan there was decreased instances of mEV colocalization around residual casein and milk protein masses (white on TEM) (Fig. 5a). Tryptophan titrations with mEVs lyophilized in 50mM TH revealed a slight decrease in zeta potential magnitude suggesting an interaction with mEV membrane surfaces beyond that of TH (Figure S2d). Tryptophan at 100µM with 50mM TH was shown to have an increased mode particle size (157.4 ± 4.4 nm) and a decreased particle standard deviation (43.6 ± 1.7 nm), with slightly increased particle counts and decreased PDI compared to 50mM TH-only groups (Fig. 5b). This distribution change seemed to be lost at low (10µM) and higher (1mM) tryptophan concentrations.

**Fig. 6:**
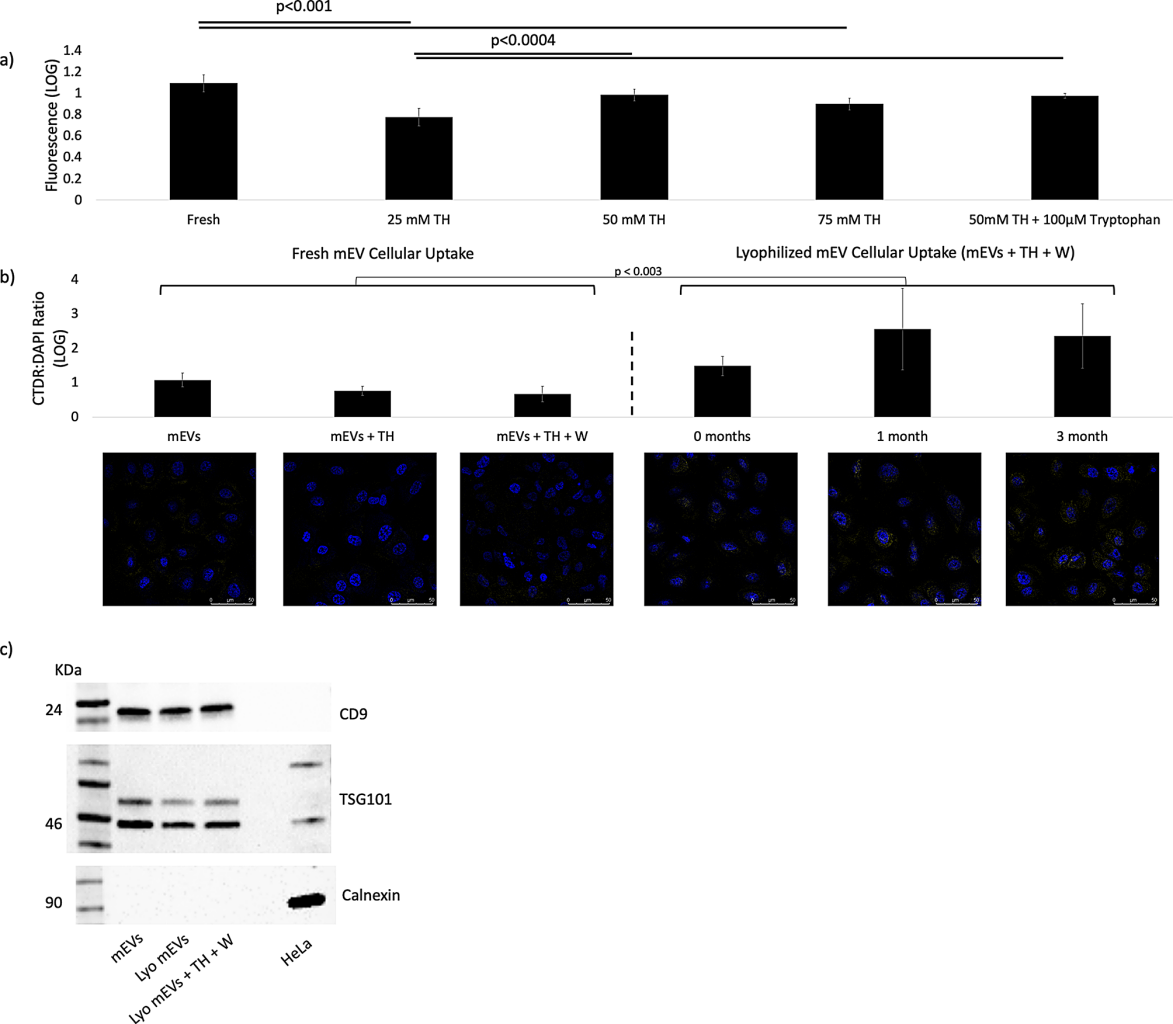
**(a)** Mean fluorescence (log scaled) of mEVs under confocal microscopy loaded with Calcein-AM post-lyophilization after removal of exogenous dye via Sepharose G50 spin column. *N* = 6 images, error bars are representative of standard deviation within each group; biological duplicates. No signal was observed in HEPES control, 10mM TH, and 50mM sucrose groups (Images in Figure S6). Multiple T tests with Bonferroni Correction (α = 0.05), reveals significant differences between fresh and 25mM TH | 75mM TH and between 25 mM TH and 50mM TH | 50mM TH + W. *N* = 6 images, 3 ROIs each **(b)** CTDR: DAPI fluorescent ratios (log-scaled) of CTDR loaded fresh mEVs supplemented with TH (50mM) and TH (50mM) + Tryptophan (W; 100 µM) on MDCK cells and 50mM TH + 100 μm Tryptophan mEVs loaded with CTDR uptake post-lyophilization over time in room temperature storage, biological triplicates with *N* = 10 pictures per sample, outliers removed. **(d)** Western blotting of lyophilized mEV isolate for CD9, TSG-101, and negative expression of Calnexin. Lyophilized samples were stored at RT for 1 month

We also saw enhanced ECIS bioactivity of mEVs supplemented with 100µM tryptophan in HuDFs following reconstitution of the freeze-dried formulation compared to fresh mEVs (*p* = 0.0048), whereas tryptophan-only controls did not show significant bioactivity (Fig. 6c). However, this enhancement of bioactivity was not significant at 1mM tryptophan, suggesting a concentration-dependent interaction between tryptophan and mEVs. This enhancement in bioactivity did not come from enhancements in thermal stability, as the Tryptophan / TH mixtures had comparable or slightly decreased T_g_ and T_m_ compared to TH-only powders (Fig. 5d). Lyophilized tryptophan-only controls (i.e. with no TH or other cryoprotectant added) did not show significant changes in the ECIS bioactivity assay (data not shown).

### mEVs retain their internal cargo, protein markers, and ability for cellular tracking post-lyophilization

While mEVs appeared to remain intact post-lyophilization on TEM, we aimed to ensure that internally loaded cargo is also protected within the mEVs. Calcein-AM is a fluorescent dye that becomes activated when exposed to esterase enzyme activity, including the endogenously existing esterase enzymes within milk mEVs [15]. By loading Calcein-AM within mEVs, lyophilizing, resuspending, and removing unloaded dye through a spin column, we are able to assess whether mEV membranes are intact post-lyophilization and if Calcein-AM signal is maintained after resuspension. We found that only 25-, 50- and 75-mM TH retained fluorescent signal post-lyophilization, while HEPES, 10mM TH and 50mM sucrose did not (Fig. 5a, Figure S4). Given that induction and maintenance of Calcein-AM fluorescence is dependent on the presence of functional esterase enzymes within the mEV, this assay also serves as a measure of EV-associate bioactivity. The addition of tryptophan did not impact Calcein-AM retention; however, this group did exhibit significantly higher variance than the other groups on imaging.

While cargo preservation is modeled by Calcein-AM retention, CTDR permanently binds to mEV membranes and other mEV constituents and can model cellular uptake of mEV particles [63]. Fresh mEV uptake in MDCK cells were largely unaffected by supplementation with 50 mM TH + 100 µM tryptophan (W) (Fig. 6b). However, lyophilized mEVs in 50mM TH + 100 µM W showed increased cellular tracking compared to their fresh counterparts, which was maintained to up to 3 months of storage at room temperature (*p* < 0.003) (Fig. 6c). Furthermore, we confirmed that two positive mEV markers (CD9, TSG101) were retained post-lyophilization and that calnexin (marker not typically found on mEVs) remained negative (Fig. 6d).

### Arginine, lysine and cysteine also enhance mEV activity in vitro

**Fig. 7:**
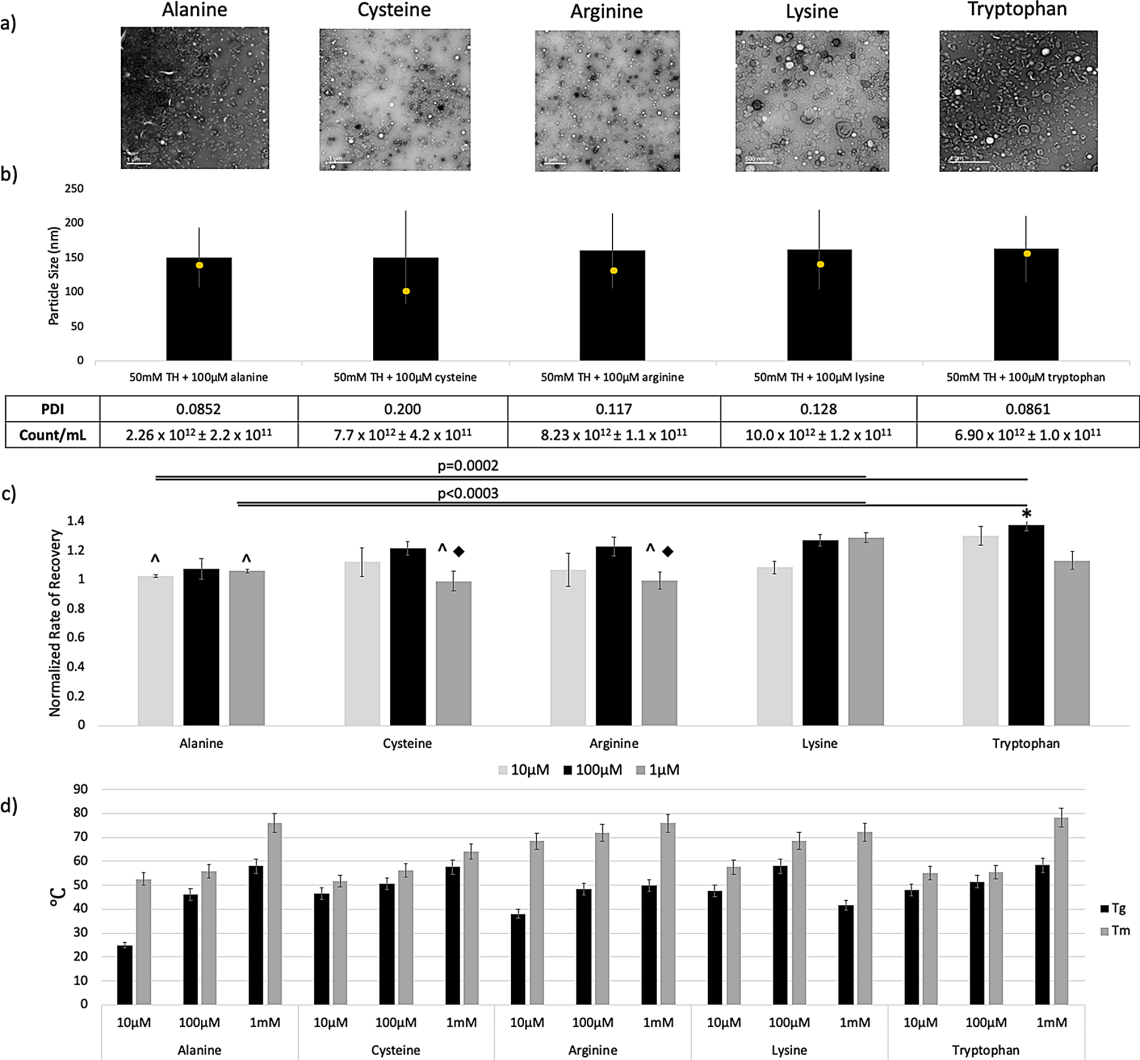
**(a)** TEM images and **(b)** NTA particle distribution (mean, mode, STDEV) of mEVs stored in 50mM TH + 100µM of amino acid **(c)** ECIS assay of bioactivity (*n* = 3) for lyophilized mEVs in 50mM TH and titrated amino acid solutions. Each well is normalized to amino-acid-only wells. Multiple T tests with Bonferroni Correction (α = 0.05) reveals 10µM and 1mM alanine samples were both statistically differed from 1mM lysine (*p* = 0.0002, 0.0003) and 100µM Tryptophan (*p* = 0.0002, 0.0002). Comparing all amino acid supplemented groups to fresh and 50mM TH groups via ANOVA with post-hoc Tukey, we find that only 50mM TH + 100µM Tryptophan had significantly improved bioactivity compared to fresh (*p* = 0.0413) and 50mM TH (*p* = 0.0386) (*); all other groups equally performed to fresh and 50mM TH or underperformed (Underperformed fresh mEVs, ^; underperformed 50mM TH lyophilized, ◆) **(d)** DSC thermal stability of mEV powders, error bars represent standard error associated with calibration

The mechanism by which tryptophan enhances mEV protection during freeze drying is not yet understood. This being said, we were interested in determining whether similar amino acids could yield comparable outcomes. Therefore, we selected several amino acids that share similar characteristics (e.g., R-group, charge, polarity) to tryptophan, including lysine (positively charged amino), arginine (guanidine R-group), cysteine (ability for particle-particle binding), and alanine as a negative “neutral R-group” control (methyl group) (Fig. 7). Other amino acids including histidine (nitrogenous imidazole R-group), tyrosine (aromatic R-group), and phenylalanine (benzyl R-group) were also tested, but showed minimal improvement over TH-only controls (Figure S5). Alanine groups performed slightly below TH-only and fresh mEV controls in all quality control metrics, suggesting that the addition of this slightly hydrophobic amino acid alone may impact mEV stability or uptake. Most notably, 1mM lysine + 50mM TH outperformed alanine in HuDF ECIS bioactivity, and both 1mM and 100µM lysine groups had notably faster recovery rates compared to controls, but these were found to be not statistically significant (*p* = 0.0017 and *p* = 0.0007, respectfully) (Fig. 7c). 100µM tryptophan was the only group to outperform fresh mEVs, suggesting a unique interaction between mEVs and indole rings.

Adding 10 µM of any amino acid decreased the T_g_ of the overall powder, likely due to disruption of homogenous TH crystals during lyophilization [54, 64]. However, T_m_ in 100 µM lysine, 1mM lysine and 1mM tryptophan increased from TH-alone to around 80ºC (Fig. 7d).

## Discussion

While many groups have investigated the therapeutic potential of mEVs, very few have reported on the impact of long-term storage and thermodynamic stress on mEV structure and function. mEVs, like many biologics, have to overcome a significant translational hurdle of maintaining stability during logistical distribution; however, unlike many biologics, mEVs cannot remain stable in solution, or in cold storage for extended periods of time, without suffering loss-of-function with respect to structure and bioactivity. Lyophilization of mEVs will facilitate commercialization by reducing the logistical challenges associated with large-scale production and storage [65].

We have shown that mEVs were reproducibly generated from bovine milk according to the protocol outlined by Marsh et al. (Fig. 1) [15], and that mEV bioactivity can be assessed leveraging ECIS, resulting in a 20% increase in HuDF monolayer recovery rates compared to controls (Fig. 2). Other groups assess EV bioactivity in a variety of different ways, mainly monitoring changes in gene expression, toxicity, ability to bind to a ligands (e.g., TNF-⍺); however, these tests typically cannot be run in parallel in a short time frame [53, 66–68]. For EV populations that are known to enhance wound recovery, our ECIS assay was able to provide reasonably reproducible, time-series migration data and a readout of bioactivity in under 20 h. With respect to this time course, it interesting to note that the effect of mEV addition appeared to occur within 2 h. Mediation of this effect via modification of gene expression or transcriptional interference (e.g. via miRNA) would not seem to provide a satisfactory causal account of this phenomenon. The Calcein-AM uptake and retention assay also provides a novel proxy for assessing mEV patency and bioactivity of a constituent enzymic activity i.e. esterases.

We have confirmed that mEVs have altered structure and bioactivity after 1 month in storage at 20ºC, and − 80ºC across all quality control metrics (Fig. 3). Storage at 4ºC and − 20ºC both retained comparable bioactivity to fresh mEVs, despite having different particle distribution means and modes. Contrary to some previously reported studies, we found that a single freeze-thaw cycle reduced both the mean and mode of nanoparticles by ~ 50 nm. Although this was found to not be statistically significant reduction, this may potentially suggest selective destruction of larger diameter mEVs during freeze-thaw events [18, 24, 29, 59, 69, 70]. The increased stability of mEVs < 150 nm may be attributed to one of several aspects including: (1) increased curvature of smaller mEVs resulting in higher surface tension and resistance to deformation or (2) increased cholesterol content in smaller mEV membranes, which as we demonstrate herein also results in higher thermal stability [71, 72].

While many groups rely heavily on particle distribution and zeta potentials to predict preservation of structure and stability, we found the small changes in zeta potential (< 5mV) does not indicate significant changes in EV stability (Figure S2). In addition, we saw that PDI obtained by DLS had extreme variability within the same sample, which may be an artifact of residual milk proteins or mEV clumping resulting in mixed scattering signals. Herein, we found that a combination of particle distribution, ability to maintain internal cargo (e.g., Calcein-AM), and ECIS bioactivity in vitro were the best predictors of functional mEVs.

While TH is a widely used excipient for lyophilization, we have found that its cryoprotective effects are concentration dependent and may be dependent on the specific EV population undergoing freeze-drying [26, 29, 73]. 50 mM TH was found to provide the best performance in maintaining structure, function and particle count, while in the absence of TH we see significant destruction of mEV nanoparticles (Fig. 3). The lyophilized powders were stable at room temperature for up to 3 months, with a T_g_ and T_m_ well over room temperature, at 40–50 °C, and was able to retain loaded Calcein-AM throughout this time (Figs. 4 and 6). Interestingly, we did observe that mEVs lyophilized with TH, once rehydrated, were not able to successfully load dye, suggesting that TH might block endogenous compounds from entering the mEV interior or that TH/mEV solutions may require a higher loading temperature than 37 °C. Additionally, it was noted that pre-SEC mEVs had a higher T_g_ and T_m_ compared to fresh controls; this may suggest that naturally occurring milk proteins and chemicals may contribute to mEV stability; however, these factors are subsequently removed during the SEC purification process. Further work is required here, as any application that requires use of mEVs as drug delivery devices and cargo loading, may be restricted by this apparent technical impediment.

We also saw that tryptophan, when added in conjunction with TH, resulted in preserved particle counts and mEV size while increasing bioactivity (Figs. 5 and 6). These effects seem to be unique to tryptophan, aside from lysine, suggesting that the nitrogenous component of the indole ring is important for mEV interaction (Fig. 7). However, this interaction is not characterized and it may be that tryptophan is interacting with residual milk proteins in solution instead of, or in addition to, interacting with the mEVs themselves. CTDR cell tracking data (Fig. 6) also suggests that tryptophan’s mechanism of action may only be occurring after lyophilization. While the mechanism is not yet known, tryptophan interactions with mEV membranes may require close proximity in order to occur (i.e., during solvent sublimation) or tryptophan may be interacting with mEVs such that they become more bioavailable to cells and become less attached to each other or exogenous proteins.

Lactadherin, a protein present in milk that has been previously reported to interact with EV membranes [61, 62], has a relatively high affinity for tryptophan, lysine, and arginine (in silico), suggesting these amino acids may be competitively inhibiting mEV-protein binding (Fig. S6). Previous crystallography studies also suggest that lactadherin’s C2 binding domain rely on tryptophan to bind with EV membrane components [74]. This interaction might also play a role in mEV during mEV transcytosis [75, 76]. Interestingly, tryptophan is one of several free amino acids present in milk, and it tends to be found in higher concentrations in colostrum compared to milk [77]. Similarly, milk extracellular vesicles (mEVs) are also more abundant in colostrum than in milk [78]. Importantly, tryptophan is considered safe for human consumption and is used as an additive in food products, as well as a supplement at concentrations that exceed the 100 µM concentration that we found most effective in increasing cryo-protection of mEVs [79].

While we did not widely investigate the impact of lyophilization parameters on mEV preservation, preliminary studies did not reveal notable differences in mEV morphology between slow, ramped freezing and vacuum compared to fast freezing at constant vacuum (Figure S1b-f). This likely suggests that formulation is more important in cryoprotection of mEVs, as opposed to lyophilization parameters. This being said, it may be that this consideration will need to be revisited as the isolation process is scaled up beyond the relatively small volumes (< 5 mL lyophilizates) investigated in this study.

Finally, while our ECIS bioactivity assay was effective at screening for bioactivity in mEVs, we must verify that these in vitro results are representative of desired in vivo actions and functions. For example, one advantage of mEVs is their proclivity for oral administration and uptake into the circulation via the gut [80]. Further work is required to confirm that the specific in vivo bioactivity is preserved following freeze-drying by the optimized formulation described here, and that the ECIS assay serves as a reliable proxy.

## Conclusion

This study demonstrates that milk-derived extracellular vesicles (mEVs) can be preserved as a stable powder at room temperature for up to 6 months without significantly impacting structure and function in vitro. The synergistic effect of TH and tryptophan, in particular, highlights the potential for further optimization of lyophilization protocols to enhance the viability and efficacy of these bioactive materials. By overcoming the critical bottleneck of maintaining EV stability outside of cold-storage environments, we pave the way for the broader adoption and commercialization of mEV-based therapies, offering a scalable, cost-effective solution for drug delivery, and beyond.

## Electronic supplementary material

Below is the link to the electronic supplementary material.

Supplementary Material 1

## Data Availability

The datasets used and/or analyzed during the current study are available from the corresponding author on reasonable request.

## Abbreviations

mEV: milk–derived extracellular vesicles
TH: trehalose
W: tryptophan
ECIS: Electric Cell Substrate Impedance Sensing
TFF: tangential flow filtration
HuDF: human dermal fibroblasts
PDI: polydispersity index
